# Anthropogenic impact is negatively related to coral health in Sicily (Mediterranean Sea)

**DOI:** 10.1038/s41598-019-49713-w

**Published:** 2019-09-17

**Authors:** Fiorella Prada, Luigi Musco, Adriana Alagna, Davide Agnetta, Eleonora Beccari, Giovanni D’Anna, Vincenzo Maximiliano Giacalone, Carlo Pipitone, Tomás Vega Fernández, Stefano Goffredo, Fabio Badalamenti

**Affiliations:** 1 Marine Science Group, Department of Biological, Geological and Environmental Sciences, University of Bologna, Via F. Selmi 3, 40126 Bologna, Italy; 2 Stazione Zoologica Anton Dohrn, Integrative Marine Ecology Department, Villa Comunale, 80121 Naples, Italy; 3 Istituto Nazionale di Oceanografia e di Geofisica Sperimentale, Via Beirut 2/4, 34151 Trieste, Italy; 4 Consiglio Nazionale delle Ricerche, Istituto per lo studio degli impatti antropici e sostenibilità in ambiente marino, Via Giovanni da Verrazzano 17, 91014 Castellammare del Golfo, Italy; 5 Consiglio Nazionale delle Ricerche, Istituto per lo studio degli impatti antropici e sostenibilità in ambiente marino, Via del Mare 3, 91021 Torretta Granitola, Italy; 6 School of Geosciences, Grant Institute, King’s Buildings, University of Edinburgh, Edinburgh, United Kingdom

**Keywords:** Biooceanography, Conservation biology, Conservation biology

## Abstract

Shallow-water marine organisms are among the first to suffer from combined effects of natural and anthropogenic drivers. The orange coral *Astroides calycularis* is a shallow-water bioconstructor species endemic to the Mediterranean Sea. Although raising conservation interest, also given its special position within the Dendrophylliidae, information about the threats to its health is scant. We investigated the health status of *A. calycularis* at five locations in northwestern Sicily along a gradient of cumulative human impact and the most probable origin of the threats to this species, including anthropogenic land-based and sea-based threats. Cumulative human impact appeared inversely related to the performance of *A. calycularis* at population, colony, and polyp levels. Sea-based human impacts appeared among the most likely causes of the variation observed. The reduction in polyp length can limit the reproductive performance of *A. calycularis*, while the decrease of percent cover and colony area is expected to impair its peculiar feeding behaviour by limiting the exploitable dimensional range of prey and, ultimately, reef functioning. This endangered habitat-forming species appeared susceptible to anthropogenic pressures, suggesting the need to re-assess its vulnerability status. Creating microprotected areas with specific restrictions to sea-based human impacts could be the best practice preserve these bioconstructions.

## Introduction

Increasing anthropogenic pressure is threatening marine biodiversity, thus impairing the provision of goods and services by coastal ecosystems to human populations worldwide^1^. Both land-based (e.g., urbanisation, agriculture, industry) and sea-based activities (e.g., fishing, shipping) exert substantial impact on marine ecosystems^2,3^. Moreover, global change may compromise survival and distribution of marine organisms since it is evident that ocean acidification, heat waves, increased frequency of extreme atmospheric events among global-change related threats, ultimately affect biodiversity and ecosystem functioning^4–7^. Global change-driven mass mortalities and disease outbreaks in coastal ecosystems are dramatically increasing^8–11^, causing natural disasters such as extreme coral bleaching events like the one that recently hit over 60% of the Great Barrier Reef^12^.

The semi-enclosed Mediterranean Sea responds faster than the global ocean to environmental change^13^. For centuries, the Mediterranean basin has been one of the areas most subject to anthropogenic pressures in the world^14^. Threats to its marine life derive from extensive coastal urbanization, shipping activities, and intense use of resources that cause pollution, eutrophication, habitat degradation, decline of fish stocks, algal blooms, spread of pathogenic organisms, and invasions by non-indigenous species, among others^13,15–17^. Thus, Mediterranean coastal ecosystems experience a combination of large-scale changes and an array of local pressures whose synergy likely impacts the marine life they support. In fact, during the last few decades mass mortalities have periodically impacted Mediterranean benthic communities after prolonged heat waves^18–22^.

In recent years, several attempts to weigh the impact of anthropogenic drivers in spatially explicit ways have been proposed at the global scale^2^. At the Mediterranean level, data on anthropogenic drivers affecting coastal systems are available, although some of them (e.g., shipping, fishing) are difficult to weigh and sometimes show low resolution levels^23,24^. Additionally, the intensity of a given driver is often assumed to be linearly related with its impact on coastal systems, but different response curves are also plausible. Moreover, estimates of anthropogenic impact on the ocean hardly take into account the possible synergy among multiple pressures, since their synergistic effect is almost impossible to test in the field beyond the local scale. These are among the main reasons why the combined potential impact of multiple pressures and drivers is often measured as the sum of the impacts in a cumulative way^2,23,24^, although synergy or antagonism among impacts may occur^25^. Micheli *et al*.^24^ identified and quantified human impacts for the Mediterranean area, producing a map of cumulative human impact by combining different drivers (both anthropogenic and climatic), having different origin (land or sea), along with their potential impact on marine habitats.

Shallow-water benthic organisms are among the first to experience anthropogenic impacts^26^. The single or combined effect of pressures can have negative consequences on vital functions of marine organisms such as reproduction, feeding and growth, ultimately leading to population declines^4,27,28^. When little is known on the biology and ecology of a species, but the assessment of its health status in the wild is required, it is almost impossible (and of limited interest) to disentangle the effects of each single driver from the others. In such cases, a holistic approach, such as the one proposed by Halpern *et al*.^2^ and Micheli *et al*.^24^, accounting for all the potential threats to biodiversity and their weight, would better depict the actual risk to the conservation status of habitats and species.

*Astroides calycularis* (Pallas, 1766) is a Mediterranean endemic shallow-water habitat-former. It is a colonial coral species typically found from the surface to 15 m depth on vertical rocky reefs, overhangs and caves^29–32^ where it can cover up to 90% of the substrates of small coves and cliffs^32^. Sparse colonies may also be found at about 50 m depth^29,33,34^. Although tropical reefs are larger and host a greater number of reef-building species^35^, Mediterranean ecosystem engineer corals such as *A. calycularis* are able to edify spatially complex rigid frameworks characterized by holes and cavities that generate numerous microhabitats hosting a rich community of associated species^32,36^, thus sharing some traits of a tropical reef^37^.

The orange coral *A. calycularis* is one of the 166 living Dendrophylliidae species worldwide^38^, this last family ranking third among Scleractinia in species richness^38,39^, and representing approximately 23% of all azooxanthellate coral species^40^. The genus *Astroides* is monospecific. Distinct microstructural features and 28S rRNA sequences^41,42^ have revealed that *A. calycularis*, and species of *Balanophyllia* and *Tubastraea* form a monophyletic group^41^, highlighting the special phylogenetic position occupied by the orange coral within the Dendrophylliidae.

*Astroides calycularis* is a gonochoric species, featuring both male and female polyps and colonies, and a brooder, releasing planulae from the tentacles during early summer^43–45^. Polyps reach sexual maturity at 3 mm length^46^. Colonies are ellipsoid and polyps in each colony may be tightly associated or separated based on the hydrodynamic regime^30,32,47^. The orange coral is an azooxanthellate species^39^ that occupies both well-lit and dark habitats with a preference for turbulent environments^32^. As other azooxanthellate corals, it is considered an obligate suspension feeder likely preying upon medium to small zooplankton, but little is known on its feeding preference. Large polyps may generally offer better opportunities for capturing large prey^48^, including sea slugs^49^, especially in azooxanthellate species that depend exclusively on food intake for their nutrition, which is in fact a common feature among the Dendrophylliidae^38^. Thus, the possible advantage of having many (relatively) small polyps, in a colonial azooxanthellate species such as *A. calycularis* remains elusive^42^. However, recent evidence suggests that *A. calycularis* preys on large gelatinous plankton using a protocooperative strategy involving polyps of several colonies^50^. This is the first coral species in which such an unusual cooperative behaviour is observed. The ability to cooperate among colonies may be the evolutionary driver for this species to form aggregations resulting in bioconstructions^50^. Consequently, densely aggregated colonies of this coral should be more effective in preying on large gelatinous plankton.

Within the Mediterranean context, *A. calycularis* is considered a thermophilic, stenotherm species i.e., thriving at relatively high temperatures, but sensitive to temperature fluctuations^51,52^, occurring in the south-western part of the Mediterranean basin from the Strait of Gibraltar to Sicily and surrounding islands^29,34,53^. It is common in southern Spain, northern Morocco, Algeria, Tunisia, and has the widest latitudinal spread in the eastern part of its range where the 14 °C isotherm reaches its northernmost expansion in the western Mediterranean Sea during winter. The latter area includes Malta and the Italian coast from Ventotene Island in the north to Lampedusa Island in the south^16,32,53,54^. Studies on the biology and ecology of *A. calycularis* have been performed mostly on populations of southern Spain and southwestern Italy, including Sicily and the Egadi Islands^36,44–46,50,52,54–57^. The coast of northwestern Sicily and of the Egadi Archipelago is located at an intermediate latitude within the species’ range, where it is expected to find optimal environmental conditions, based on the assumption that at the center of its geographical range, a species is generally favored and tends to show the highest population density^58^ (and references therein). In fact this area forms one of the main hotspots of distribution and abundance for *A. calycularis*^29,53^ and appears a good candidate location for studying the biology and ecology of this species^32,45,50,53,56,59^.

Anthropogenic threats to this species are not fully defined yet. While pollutants - whose effects are still unknown - may pose risks^60^, increased temperature and ocean acidification show negative effects on *A. calycularis* survival and net calcification rate^34,61,62^. Also, recreational diving has shown detrimental effects on this species. Colonies of *A. calycularis* laying on the seabed after detachment by unintentional physical contact by divers are common in crowded diving points^34,56,59^. However, it can be reasonably hypothesized that other stressors may threaten this species. As observed for other Mediterranean coral species and coralligenous formations^63,64^, exploitation of sea resources (e.g. fishing), the spread of non-indigenous species, coastal overpopulation and associated forms of pollution (e.g. fertilizers, pesticides, urban runoff) can potentially determine human impact at various levels of biological organization. In fact, smothering of coral polyps due to suspended particulate organic matter and sedimentation may lead to reduced linear extension^3,65^, while high levels of nitrogen may impair reproduction and larval development^66,67^. Increased sedimentation may also affect coral health, leading to detrimental effects on polyp tissues, enhancing bacterial infections, reducing recruitment and settlement of coral larvae, and inhibiting growth thus reducing life span^68,69^. Moreover, increased terrestrial runoff and direct effects of fishing gears have been linked to severe reductions in coral cover on a global scale^70–73^. It is expected that human impact may impair the performance of *A. calycularis* at increasing levels of biological organization, from polyp to colony to population.

Despite being included as an endangered/threatened species in the UNEP protocol on the biological diversity in the Mediterranean^73^ and by national and international conventions (Berna and Barcelona Conventions, CITES), the International Union for Conservation of Nature (IUCN) has listed *A. calycularis* as Least Concern. Each species has a unique evolutionary history that can be quantified for conservation purposes^74^. Thus, given its relatively restricted distribution, the anthropogenic and natural threats potentially affecting its populations, especially those in shallow-waters, and its unique phylogenetic position, the conservation status of this species needs to be re-evaluated.

The aim of this study is to estimate the health status of the orange coral *A. calycularis* along a gradient of human impact, in order to get valuable baseline information on the risks posed to the conservation of this habitat former. In particular, we aimed at 1) investigating the health status of *A. calycularis* at increasing level of biological organization (polyp, colony, population) at five locations along a gradient of cumulative human impact, and 2) identifying the main origin of the threats to the species and its bioconstructions.

## Methods

### Study area and assessment of impacts

**Figure 1:**
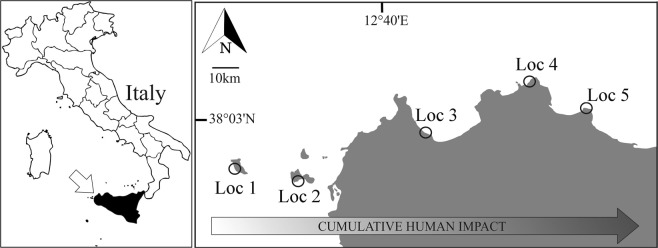
Map of the study area. Five locations are ordered following the increasing gradient of cumulative human impact. Loc 1 = Marettimo (37°58′60.00″N; 12°3′54.08″E); Loc 2 = Favignana (37°54′43.00″N; 12°17′55.00″E); Loc 3 = Zingaro (38°5′39.90″N; 12°48′17.07″E); Loc 4 = Capo Gallo (38°12′8.71″N; 13°15′59.41″E); Loc 5 = Capo Zafferano (38°7′9.50″N; 13°30′31.25″E).

The study was conducted at five locations lying approximately at the same latitude (between 37°N and 38°N) on the north-western coast of Sicily, western Mediterranean. The study area is characterized by a gradient of increasing urbanization moving eastward from the Egadi Archipelago to the Gulf of Palermo (Fig. 1). Five locations tens of kilometres apart, characterized by increasing levels of anthropogenic pressure were randomly selected among those hosting well-developed populations of *A. calycularis* after a preliminary survey (Fig. 1). Locations, according to a scale from minimum to maximum pressure, included: Marettimo (Loc 1) and Favignana (Loc 2) Islands (both inside the Egadi Islands marine protected area), the Zingaro coast (Loc 3) (whose land part is a nature reserve), Capo Gallo (Loc 4) (within the municipality of Palermo, located inside the Capo Gallo-Isola delle Femmine marine protected area, at the westernmost point of the Gulf of Palermo), and Capo Zafferano (Loc 5) (at the easternmost point of the Gulf of Palermo). The five sampling locations were all situated on vertical or subvertical calcareous cliffs (Permission No. 2220/2014 AS, Egadi MPA; Permission No. 18 13/10/2014 Capo Gallo-Isola delle Femmine MPA; Communication to Coast Guard Authorities No. 0004795 20/05/2014 – IAMC-CNR).

At each location, human impact was assessed adapting the method proposed by Micheli *et al*.^24^ to the specific case study. Among the agents of change reported by these authors, the following were considered as potentially impacting *A. calycularis*: human population, fertilizers, pesticides, urban runoff, shipping, invasive species, artisanal fishing and four types of commercial fishing (destructive demersal, demersal with low by-catch, demersal with high by-catch, pelagic). In order to obtain the matrix of pressures of the above mentioned agents for each location, the value of each agent of change at each location was extracted using ArcGis 9.3 from the georeferenced layers, 1 km^2^ resolution, developed by Micheli *et al*.^24^. The raw matrix thus obtained was then standardized to make each response variable (agent of change) vary within the same scale, from 0 to 1. This was achieved by dividing the scores of the agent at the five locations by the maximum value obtained for such given agent across the five studied locations. The obtained standardized matrix allowed for relative comparisons of the importance of the different agents across the study area.

Subsequently, the potential effect of each agent of change on *A. calycularis* was elicited from experts on the biology of the species and the study area. Experts, who were not aware of the experimental design and data, were asked to rank the importance of each agent of change along a scale of discrete values between 0 (not important at all) and 10 (very important). For each agent, the average score was obtained after reducing the variability of judgments, by excluding *a posteriori* the minimum and maximum value (outliers) for each variable (if two or more experts expressed the same outlier judgment, only one of the values was excluded).

The estimated impact of each agent of change on the studied species was calculated by multiplying the standardized value of the pressure exerted by a given agent at a given location (from the GIS layers by Micheli *et al*.^24^) by its average importance score across the elicited experts. For example, if the standardized value of urban runoff at location 1 was 0.5, and the average expert judgment for that agent was 7, the impact of urban runoff on the species was 0.5 ∗ 7 (see Supplementary Table S6). The cumulative human impact (*Ic*) for each location was obtained by summing up the estimated impact values for the different agents of change. *Ic* was partitioned into sea-based (*I*_*S*_) and land-based (*I*_*L*_) human impacts, so that *I*_*C*_ = *I*_*L*_ + *I*_*S*_ at any given location. *I*_*S*_ was obtained as described above considering shipping, invasive species, artisanal fishing and four types of commercial fishing. *I*_*L*_ was obtained considering human population, fertilizers, pesticides, and urban runoff. The obtained *I*_*C*_, *I*_*L*_ and *I*_*S*_ values are shown in Supplementary Table S2. *I*_*C*_ and *I*_*L*_ increased along the west-east direction (Fig. 1; see Supplementary Table S2), while *I*_*S*_ increased along the west-east direction from Loc 1 to Loc 4, while Loc 5 showed an *I*_*S*_ value lower than Loc 3 and Loc 4, but higher than Loc 1 and Loc 2.

The five locations were chosen within the narrowest latitudinal range possible in order to reduce potential effects of natural environmental variation on the performance of this stenotherm species. Nonetheless, we also tested the potential correlation with average sea surface temperature (SST AVG) and average annual variation of sea surface temperature (SST VAR), i.e. average difference between maximum and minimum annual temperatures. Five-year temperature data (of the five years preceding the sampling: 2009 to 2013) were extracted from the NASA online application Giovanni (https://giovanni.gsfc.nasa.gov/giovanni/). SST AVG increased along the west-east direction form 19.78 °C at Loc 1 to 20.55 °C at Loc 5; SST VAR varied from 12 °C at Loc 2 to 13.51 °C at Loc 5 (Supplementary Table S3).

### Biological response variables

Fieldwork was carried out between May and July 2014. In order to assess the health status of the species along the cumulative impact gradient and to check it at multiple levels of biological organization, three response variables were considered. The percent cover, considered an important indicator of health status in coral reef studies^75,76^, was analyzed as a proxy of health at the population level. Colony area and polyp length were selected as proxies of health at the colony and polyp levels, as these biometric parameters have been used in biometric, reproductive biology, and population dynamic studies of several temperate coral species^55,77^ as a measure of size, which is a good proxy of health in long‐lived marine invertebrates like *A. calycularis*^55,77,78^.

### Percent cover

**Figure 2:**
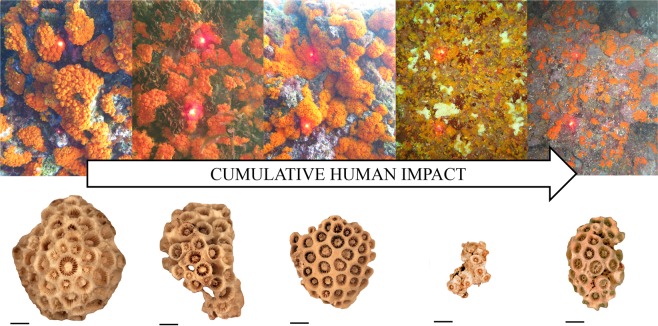
*Astroides calycularis* cover (top) and, morphology (bottom) along a gradient of increasing Cumulative Human Impact. From the left to the right: Loc 1 = Marettimo; Loc 2 = Favignana; Loc 3 = Zingaro; Loc 4 = Capo Gallo; Loc 5 = Capo Zafferano. Red dots in each upper frame represent the reference scale (20 cm). Scale bar (bottom) is 1 cm. Pictures were subjectively selected to represent the observed condition at each study location.

The percent cover of *A. calycularis* was quantified in three sites hundreds of m apart within each location, at depths between 1 and 3 meters. Sites were chosen in order to represent the optimum condition for the population in the analyzed location (i.e., where the species appeared more abundant). At each site, percent cover was determined by taking six photos, which acted as replicates, on vertical/sub-vertical walls in order to avoid any confounding effect of substrate slope on the response variables. The camera was equipped with two laser pointers set at a fixed 20 cm distance, which produced a reference scale for each picture (Fig. 2). The digitizing software Image J was used to calibrate each photo setting the reference distance given by the two pointers and to measure the area occupied by *A. calycularis* colonies inside 25 × 25 cm^2^ quadrats by tracing their outline with a hand-controlled mouse on the digital image.

### Colony and polyp biometry

Three to five colonies of *A. calycularis* were collected in the same sites used for the analysis of percent cover. The sampled colonies were randomly chosen among those settled within previously photographed frames. In each location, samples were collected from the three sites, except for Loc 1 and Loc 2 where only two sites were sampled for logistic reasons. The collected colonies were kept immersed in 10% sodium hypochlorite for 10 days to remove the soft tissue, then dried at 50 °C for 4 days. Skeletons were observed under a binocular microscope to remove fragments of substratum and calcareous deposits produced by epibionts. The length (L_C_: major axis of the colony) and width (W_C_: minor axis of the colony) were measured with a calliper in order to obtain the colony area that was calculated using the formula for the ellipse ${\rm{A}}=\frac{\pi \,\ast \,({{\rm{L}}}_{{\rm{C}}}\,\ast \,{{\rm{W}}}_{{\rm{C}}})}{4}$. Colony area was chosen as the main biometric parameter for colonies as it is considered a more accurate and representative measure of colony size than colony length^79–82^.

The number, length (i.e., maximum axis of the oral disc) and width (i.e., minimum axis of the oral disc) of all polyps was determined for each colony^55^. Polyp length was selected as a proxy of polyp size. The distribution of polyp size classes at each location was then determined by considering 1 mm as the threshold between a size class and the subsequent one^55^.

### Statistical analyses

The proportions of variation of (1) cover, (2) colony area, and (3) polyp length explained by *I*_*C*_, *I*_*L*_, *I*_*S*_, SST AVG and SST VAR were checked by non-parametric multiple regression analyses using the DISTLM-forward procedure (distance-based multivariate analysis for fitting a linear model using forward selection of factors). The analyses were performed using the software PRIMER v6 including the PERMANOVA + add-on package^83^.

Differences in the frequency distribution of polyp size classes among locations were tested by means of a Kruskal-Wallis test. Where significant, pairwise comparisons between locations were performed via Kolmogorov-Smirnov tests.

## Results

Overall, considering the results of all the DISTLM tests, *I*_*C*_, *I*_*L*_, SST AVG and SST VAR all appeared highly correlated between each other (R > 0.78; Supplementary Table S4). Thus, disentangling the relative portions of explained variation of each variable in the study area was not possible, while *I*_*S*_ resulted as the most independent predictor variable (Supplementary Table S4).

### Percent cover

**Figure 3:**
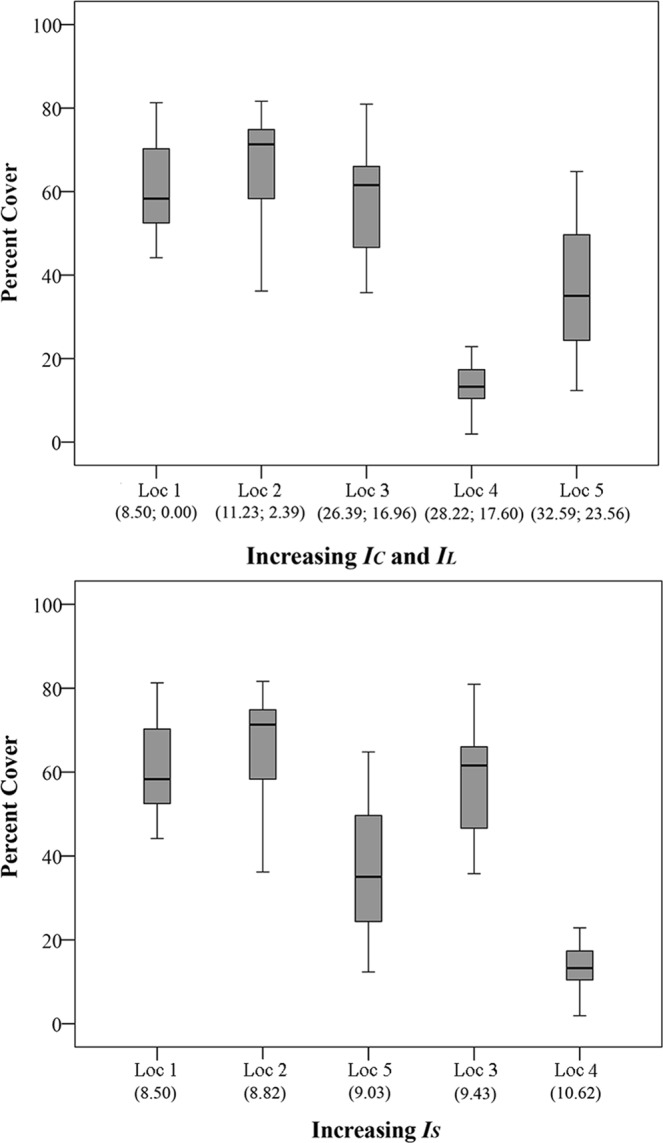
*Astroides calycularis* percent cover at five locations along gradients of Cumulative, Land-based and Sea-based Human Impacts. Top panel: In brackets, Cumulative **(*IC*)** and Land-based **(*IL*)** human impact values, respectively. Bottom panel: In brackets, Sea-based human impact values (*IS*). Loc 1 = Marettimo; Loc 2 = Favignana; Loc 3 = Zingaro; Loc 4 = Capo Gallo; Loc 5 = Capo Zafferano. Median = horizontal line; 25th and 75th percentiles = vertical boxes; 10th and 90th percentiles = whiskers. n = 18 for each location.

The percent cover of *A. calycularis* decreased by ~38% between the two extremes of the cumulative human impact gradient from Loc 1 (low *I*_*C*_) to Loc 5 (high *I*_*C*_) (Figs 1–3).

The DISTLM marginal tests revealed that *I*_*S*_ significantly explained ~53% of the observed variation followed by SST AVG and SST VAR (~39% each) and *I*_*L*_ (~33%) (Table 1). Given the above mentioned high correlation among predictor variables, the DISTLM sequential test, fitting *I*_*C*_, SST AVG and SST VAR within the same model, revealed that SST VAR and SST AVG significantly explained ~39% and ~12% of the observed variation respectively, while the test for *I*_*C*_ was not significant after the forward selection procedure (Table 2). The DISTLM sequential test, fitting *I*_*L*_, *I*_*S*_, SST AVG and SST VAR within the same model, revealed that *I*_*S*_ significantly explained ~53% of the observed variation followed by SST VAR (~11%), *I*_*L*_ (~8%) and SST AVG (~2%) (Table 3).

**Table 1:** Non-parametric multiple regression analysis.

	Marginal Tests				
		SS(trace)	Pseudo-F	P	Prop.
Percent cover	*I*_*C*_	1.88	43.99	**0.001**	0.33
	*I*_*L*_	1.68	37.46	**0.001**	0.30
	*I*_*S*_	2.98	98.85	**0.001**	0.53
	SST AVG	2.17	55.25	**0.001**	0.39
	SST VAR	2.18	55.53	**0.001**	0.39
Colony area	*I*_*C*_	686.22	9.12	**0.004**	0.19
	*I*_*L*_	615.68	7.99	**0.006**	0.17
	*I*_*S*_	896.06	12.83	**0.003**	0.25
	SST AVG	396.82	4.80	**0.025**	0.11
	SST VAR	143.24	1.61	0.228	0.04
Polyp lenght	*I*_*C*_	735.03	255.74	**0.001**	0.10
	*I*_*L*_	681.59	235.30	**0.001**	0.09
	*I*_*S*_	971.11	350.03	**0.001**	0.13
	SST AVG	682.75	235.74	**0.001**	0.09
	SST VAR	479.95	160.95	**0.001**	0.06

DISTLM-forward analysis marginal tests independently testing correlation between Cumulative (*I*_*C*)_), Land-based (*I*_*L*_) and Sea-based (*I*_*S*_) human impact, average sea surface temperature (SST AVG), average annual variation of sea surface temperature (SST VAR) and *Astroides calycularis* percent cover, colony area, and polyp length. SS(trace) = sum of squares, Pseudo-F = Statistics, P = probability, Prop. = Proportion of explained variation. Significant P-values in bold.

**Table 2:** Non-parametric multiple regression analysis.

	Sequential Tests				
		SS(trace)	Pseudo-F	P	Prop.
Percent cover	SST VAR	2.18	55.53	**0.001**	0.39
	*I*_*C*_	0.13	3.28	0.061	0.02
	SST AVG	0.69	22.33	**0.001**	0.12
Colony area	*I*_*C*_	686.22	9.12	**0.005**	0.19
	SST VAR	211.35	2.95	0.103	0.06
	SST AVG	0.80	0.01	0.899	0.00
Polyp lenght	*I*_*C*_	735.03	255.74	**0.001**	0.10
	SST VAR	7.11	2.47	**0.124**	0.00
	SST AVG	0.27	0.09	0.765	0.00

DISTLM-forward analysis sequential tests fitting cumulative human impact (*I*_*C*_), average sea surface temperature (SST AVG) and average annual variation of sea surface temperature (SST VAR) in the same model in order to analyze their combined contributions in explaining the variation of *Astroides calycularis* percent cover, colony area, and polyp length in the study area. Analysis based on 9999 permutations of data/residuals. SS(trace) = sum of squares, Pseudo-F = Statistics, P = probability, Prop. = Proportion of explained variation. Significant P-values in bold.

**Table 3:** Non-parametric multiple regression analysis.

	Sequential Tests				
		SS(trace)	Pseudo-F	P	Prop.
Percent cover	*I*_*S*_	2.98	98.85	**0.001**	0.53
	SST VAR	0.62	26.58	**0.001**	0.11
	SST AVG	0.12	5.26	**0.033**	0.02
	*I*_*L*_	0.45	26.31	**0.001**	0.08
Colony area	*I*_*S*_	896.06	12.83	**0.003**	0.25
	*I*_*L*_	138.98	2.04	0.145	0.04
	SST VAR	201.89	3.13	0.080	0.06
	SST AVG	7.80	0.12	0.722	0.00
Polyp lenght	*I*_*S*_	971.11	350.03	**0.001**	0.13
	SST AVG	128.81	47.34	**0.001**	0.02
	SST VAR	0.79	0.29	0.592	0.00
	*I*_*L*_	1.77	0.65	0.425	0.00

DISTLM-forward analysis sequential tests fitting land-based human impact (*I*_*L*_) sea-based human impact (*I*_*S*_), average sea surface temperature (SST AVG) and average annual variation of sea surface temperature (SST VAR) in the same model in order to analyze their combined contributions in explaining the variation of *Astroides calycularis* percent cover, colony area, and polyp length in the study area. Analysis based on 9999 permutations of data/residuals. SS(trace) = sum of squares, Pseudo-F = Statistics, P = probability, Prop. = Proportion of explained variation. Significant P-values in bold.

### Colony and polyp biometry

**Figure 4:**
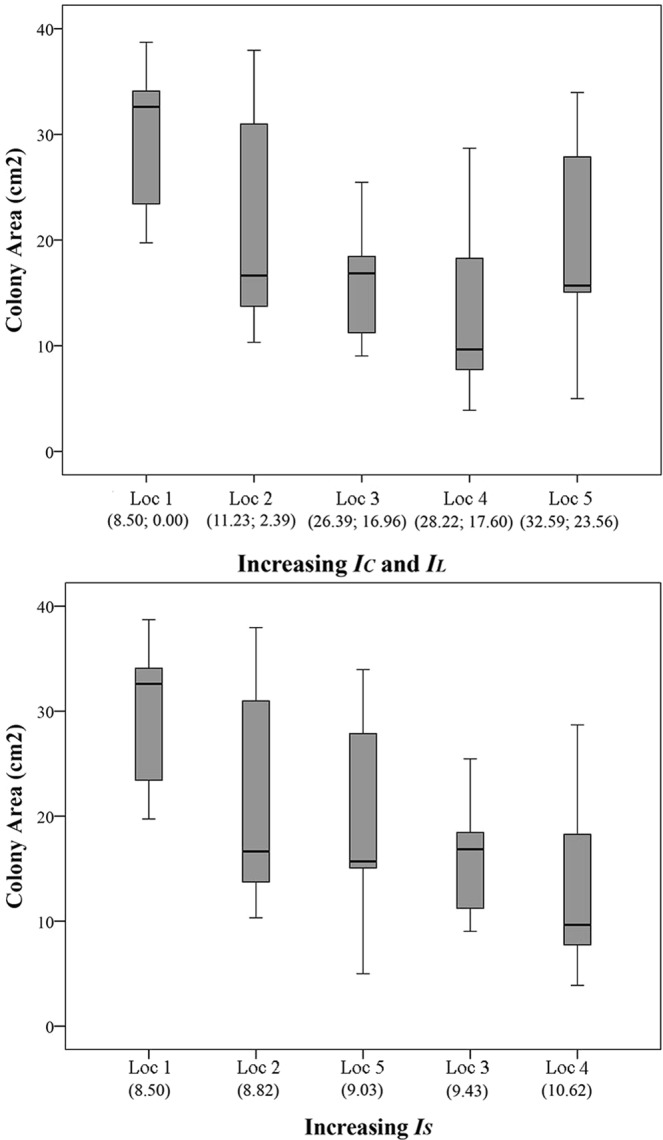
*Astroides calycularis* colony area at five locations along gradients of Cumulative, Land-based and Sea-based Human Impacts. Top panel: In brackets, Cumulative **(*IC*)** and Land-based **(*IL*)** human impact values, respectively. Bottom panel: In brackets, Sea-based human impact (***IS***) values. Loc 1 = Marettimo; Loc 2 = Favignana; Loc 3 = Zingaro; Loc 4 = Capo Gallo; Loc 5 = Capo Zafferano. Median = horizontal line; 25th and 75th percentiles = vertical boxes; 10th and 90th percentiles = whiskers. Loc 1, n = 6; Loc 2, n = 6; Loc 3, n = 11, Loc 4, n = 9; Loc 5, n = 9.

Colony area decreased by ~36% between the two extremes along the gradient of *I*_*C*_, from Loc 1 to Loc 5 (Figs 2 and 4).

*I*_*S*_ explained ~25% of colony area variation according to the DISTLM marginal test, followed by *I*_*C*_ (~19%), *I*_*L*_ (~17%), SST AVG (~11%), while the test for SST VAR was not significant (Table 1). The DISTLM sequential test, fitting *I*_*C*_, SST AVG and SST VAR within the same model, revealed that *I*_*C*_ significantly explained ~19% of the observed variation, while the tests for SST VAR and SST AVG were not significant (Table 2). The DISTLM sequential test, fitting *I*_*L*_, *I*_*S*_, SST AVG and SST VAR within the same model, revealed that *I*_*S*_ significantly explained ~25% of the observed variation while the tests for the other variables were not significant (Table 3).

**Figure 5:**
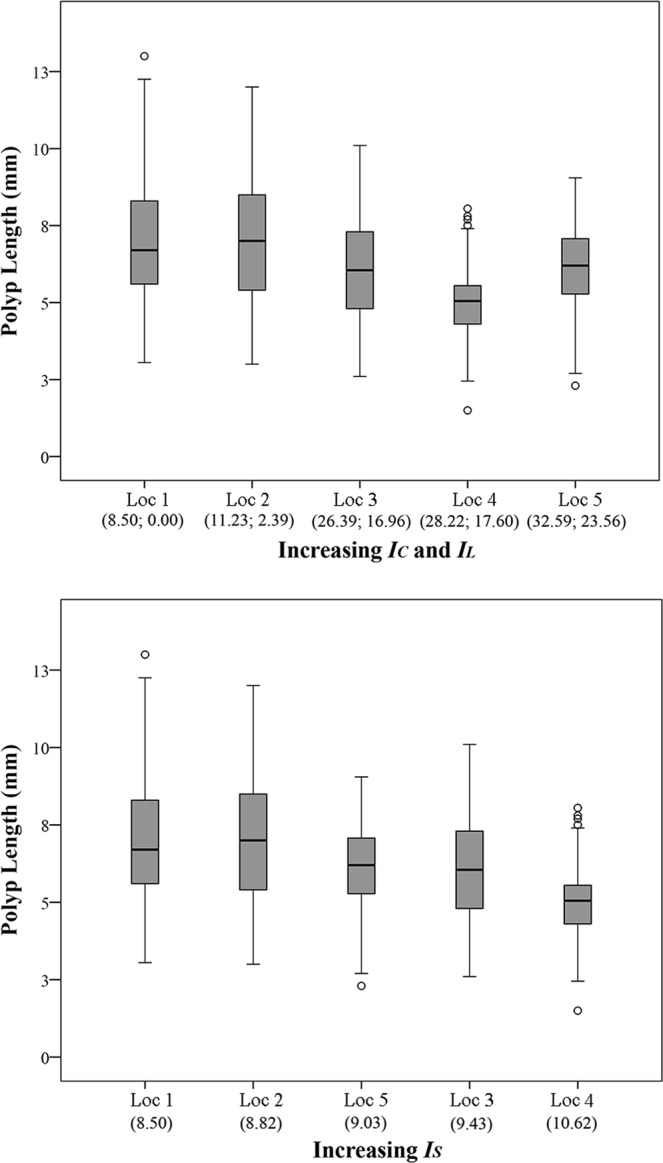
*Astroides calycularis* polyp mean length at five locations along gradients of Cumulative, Land-based and Sea-based Human Impacts. Top panel: In brackets, Cumulative **(*IC*)** and Land-based **(*IL*)** human impact values, respectively. Bottom panel: In brackets, Sea-based human impact (***IS***) values. Loc 1 = Marettimo (n = 642); Loc 2 = Favignana (n = 442); Loc 3 = Zingaro (n = 449); Loc 4 = Capo Gallo (n = 300); Loc 5 = Capo Zafferano (n = 535). Median = horizontal line; 25th and 75th percentiles = vertical boxes; 10th and 90th percentiles = whiskers.

Similarly to colony area, polyp length decreased from Loc 1 to Loc 5 (Fig. 5). According to the DISTLM marginal test, *I*_*S*_ ranked first among the predictor variables, explaining ~13% of the variation, followed by *I*_*C*_ (~10%), *I*_*L*_ and SST AVG (~9%), and SST VAR (~6%). The DISTLM sequential test, fitting *I*_*C*_, SST AVG and SST VAR within the same model, revealed that *I*_*C*_ explained ~10% of the observed variation, while the tests for the other variables were not significant (Table 2). The DISTLM sequential test, fitting *I*_*L*_, *I*_*S*_, SST AVG and SST VAR within the same model, revealed that *I*_*S*_ and SST AVG significantly explained ~13%, and ~2% of the observed variation respectively, while the tests for SST VAR and *I*_*L*_ were not significant (Table 3).

**Figure 6:**
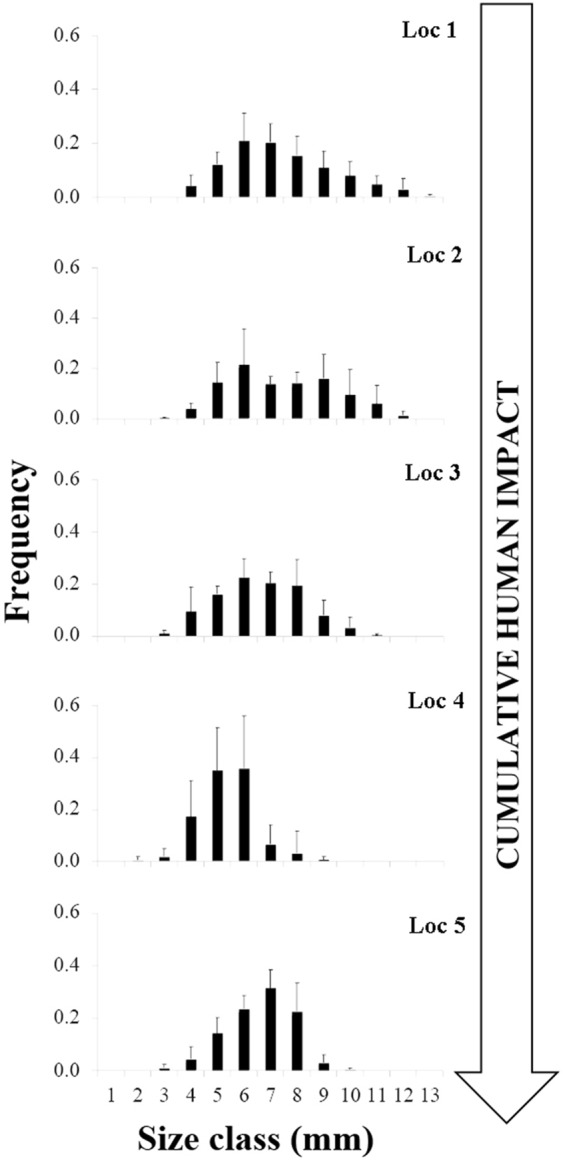
Frequency of *Astroides calycularis* polyp size classes at five locations along a gradient of cumulative human impact. Loc 1 = Marettimo (n = 642); Loc 2 = Favignana (n = 442); Loc 3 = Zingaro (n = 449); Loc 4 = Capo Gallo (n = 300); Loc 5 = Capo Zafferano (n = 535).

Polyp size classes ranged from 2 to 13 mm. The minimum number of polyps measured in a colony was 13 (at Loc 4), the maximum was 134 (at Loc 1). The gradient of human impact had an effect on the median of the size distribution of polyps among locations (H_4_ = 320.64, p < 0.001, n = 2367). Pair-wise comparisons showed that the size frequency distributions of polyps were not significantly different between Loc 1 and Loc 2, while they differed among the remaining localities, and among these and the former two (see Supplementary Table S1). Following the gradients of *I*_*C*_, *I*_*L*_, and *I*_*S*_, the observed pattern of distribution of polyp size classes appeared coherent with the *I*_*S*_ gradient in the study area, with Loc 1 and Loc 2 showing comparable *I*_*S*_ values (8.5 and 8.8 respectively), higher than those observed at Loc 3 and Loc 5 (9.4 and 9 respectively), while the highest *I*_*S*_ value (10.6) was found at Loc 4 (see Supplementary Table S2, Fig. 6). Average polyp length, standard deviation and number of size classes were higher at Loc 1 and Loc 2, and lowest at Loc 4 and Loc 5 (see Supplementary Table S5). The modal class ranged from 6 to 7 mm, except for Loc 2 where the size distribution had two modes at 6 and 9 mm. Skewness was generally positive except for Loc 5, with absolute maximum values at Loc 1 and Loc 5. Kurtosis was generally negative except for Loc 4 where size classes were fewer and frequencies around the mode dropped abruptly, particularly to the right end of their distribution (Fig. 6).

## Discussion

Anthropogenic pressure negatively relates to the health status of *A. calycularis* at all the organization levels here considered, from polyp to population. Our research suggests that both land- and sea-based threats impact the species performance, although local variation in sea surface temperature may have a role in explaining the observed pattern. However, the linearly decreasing trend in *I*_*S*_ recorded in the study area had a stronger explanatory power than *I*_*L*_, *I*_*C*_, and SST spatial-temporal variation in describing the patterns observed at all organization levels.

In this study, *I*_*S*_ showed a stronger relationship with percent cover, explaining more than 50% of its variability. The aim of this study was not to identify the weight of every single agent of change, as the study was not designed for this purpose. However, it is interesting to note that artisanal (i.e., inshore) fishing effort appeared strongly related with the reduction in *A. calycularis* cover along the analyzed gradient (data not shown). Inshore fishing is common in the investigated area, and nets thrown in proximity of the rocky substrate might damage the colonies. Professional and recreational fishing activities, as well as lost fishing gear have been shown to negatively affect not only the abundance but also the morphological and mechanical characteristics of Mediterranean hard-bottom communities^29,84,85^. Entanglement on habitat-forming macro-invertebrates, such as corals, damage their structures through mechanical friction, thus favouring parasitic colonization of the exposed skeleton ultimately leading to death^86^. However, the most likely explanation for the observed correlation in *A. calycularis* could be that fishing and *I*_*S*_ in general could represent proxies of a generalized overexploitation in densely populated areas, thus increasing the possibility of accidental contacts and subsequent damage or even detachment of the colonies. Recreational, and often illegal harvesting of shallow-water sea urchins, abalones and other traditional seafood, as well as spearfishing, are common activities in the study area. These activities often imply direct human contact with the reefs and sometimes the illegal use of poison (e.g., copper sulphate to catch octopuses), as reported by local fishermen. The reduction in percent cover by ~38% along the cumulative human impact gradient could also influence the orange coral bioconstructions at a functional level. Polyps belonging to neighbouring colonies are known to cooperate to catch large prey, and it has been hypothesized that this behaviour may explain the tendency of this species to aggregate and form biogenic reefs^50^. If aggregation enhances the ability of this species to catch large jellyfish, the reduction of percent cover in impacted areas is expected to limit *A. calycularis* ability in capturing such prey, affecting its nourishment and fitness and, ultimately, impairing an ecological function of the biogenic reefs. Following this hypothesis, such indirect, potential effect of human impact could be widespread among other cnidarians. In fact predatory cooperation among polyps has been observed in other gregarious/colonial species^87–89^.

Besides percent cover, also colony area and polyp length significantly decreased by ~36% and ~13% respectively along the gradient (Figs 4 and 5). *I*_*S*_ showed significant relations with these morphometric variables. Additionally, polyp size frequency distributions at the most impacted locations showed a decrease in the number of size classes with a lack of the largest ones, and significant transitions of size, suggesting a reduction in recruitment efficiency^90^. Also, the colony with the largest number of polyps was found at Loc 1 (N = 134), while the smallest ones were found at Loc 4 (N = 13) and Loc 5 (N = 19). Thus, the observed decline in the biotic variables is a likely effect of the overexploitation of the coastal area previously discussed for percent cover. Also, mechanical damage with accidental removal of colonies in highly impacted areas, besides reducing the overall coral cover, may cause the reduction in number of larger colonies in favour of new, smaller ones that would grow in the space made available by the damaged colonies. This would also explain the reduction of polyp length as well as the reduction in the number of size classes along the gradient, since larger polyps are usually found at the centre of larger colonies^43^.

However, the possible contributions of land-based human impact and of spatial temporal variation of SST in the study area should not be overlooked. As far as the SST and its annual variation are concerned, it is well known that *A. calycularis* is a thermophilic stenotherm species and large temperature fluctuations might impair its performance^16^. In the study area, the largest difference in average annual SST among locations was about 1 °C, while the difference among Loc 1 (Marettimo) and Loc 4 (Capo Gallo), where the species showed the best and the worst performance, respectively, was 0.66 °C. Annual SST variation ranged from 12 °C at Loc 2 to 13.51 °C at Loc 5, but at Loc 4 it was only 0.47 °C higher than Loc 1. Winter minimum SST below 14 °C is known to limit the distribution of this species^16^, but this is not the case of the study area. Moreover, *A. calycularis* had its best performance at Loc 1 and Loc 2 where the lowest minimum SST occurred. The highest maximum SST were detected at Loc 4 and Loc 5, which were about 1 °C higher than at Loc 1. It should also be noted that the highest summer average SST detected in the study area (about 28 °C in August at Loc 5) is similar to other more southern areas where *A. calycularis* thrives (e.g. the Pelagie Archipelago)^53^. In summary, although SST and its annual variation may be related to the health status of the species in the study area, it is unlikely that they may have had a leading role in determining the observed variation in *A. calycularis* health status. The observed high correlation among SST AVG, SST VAR, *I*_*c*_ and *I*_*L*_ could likely be spurious.

As far as the land-based human impact is concerned, the coastal area between Loc 4 and Loc 5 occurs within the Gulf of Palermo, which is among the most densely populated areas in Sicily (above 4000 citizens per km^2^). This area is characterized by the presence of rivers that have been proved to discharge xenobiotic contaminants to the sea (e.g., chromium-enriched sediments found in river estuaries)^91^. The Palermo area hosts a variety of industrial, agricultural, commercial and above all harbor-related activities, with the latter considered the main local source of contaminants for the coastal zone^91,92^. Increasing levels of contaminants (e.g., polycyclic aromatic hydrocarbons - PAHs), far exceeding national and international regulatory guidelines have been detected in the area^93^. The population density of coralligenous species, including corals, decreases with increasing pollution^94^ and in general pollution due to metals and organic compounds is detrimental for the marine biota^95,96^. For example, orthophosphate ions (used in fertilizers) inhibit calcification^97^, which may be heavily detrimental for stony corals. Thus, toxic metal compounds or fertilizers that reach the seawater could have affected the fitness of *A. calycularis* at the more impacted locations, contributing in part to explain the observed patterns. Further studies are needed to test these hypotheses.

Whatever the cause, the overall effect of increasing human impact is the reduction of the species performance at all the considered organizations levels. The regeneration potential and adaptability of corals^98,99^ are reflected in their ability to display, in some cases, a mixed mode of sexual and asexual reproduction^100^. Simultaneous mixed reproduction can help organisms to adapt to changing environments^101^ and can be seen as a “best-of-both-worlds” scenario. Generally speaking, in a stable environment asexual reproduction is employed to exploit suitable conditions for survival as all the new individuals will be adapted to that environment^102^. However, when environmental conditions are less favourable sexual reproduction is expected, as it ensures a mixing of the gene pool, leading to genetically diverse offspring that can exploit a broader spectrum of resources and adapt to a broader range of environmental conditions^102^. Fecundity of *A. calycularis* (i.e., the number of mature oocytes produced per female polyp) is known to decrease with decreasing polyp and colony size^43^. Consequently, we can hypothesize that at the less favourable sites *A. calycularis* could allocate more energy toward sexual rather than asexual reproduction, as expected from a species facing adverse environmental conditions. Moreover, the limited amount of offspring produced by smaller colonies and polyps at disturbed locations could not be sufficient to increase the percent cover to a level comparable to less impacted sites. This might contribute to explain the fragmented pattern of distribution observed in Loc 4 and Loc 5 (Fig. 2), with small and dispersed colonies and low percent cover, as opposed to the larger colonies found in Loc 1 and Loc 2 that colonize the available space by polyp budding, resulting in a higher percent cover. Further studies on the ecology and reproductive biology of *A. calycularis*, also during its earliest life stages, are needed to test these hypotheses.

## Conclusion

Despite the ‘vulnerable’ status recognized by several international conventions (Bern Convention [Annex II]; Barcelona Convention [Annex II]; CITES Convention), *A. calycularis* is categorized as a Least Concern species by the IUCN Committee. Nonetheless, this study has shown that population abundance and biometry at the colony and polyp levels in this species are severely impacted by anthropogenic pressure. Given the importance of this species as a bioconstructor and habitat former, and as an attraction to recreational divers, who represent an important source of income for coastal tourism, the current vulnerability status of *A. calycularis* should be re-assessed, and dedicated management plans should be considered. In the Egadi Archipelago diving is an important tourist activity with eight diving centers in the area. Diving is common between May and October with a peak between July and August with about 40 dives per day in Favignana, 10 in Levanzo and 80 in Marettimo, with 85–90% of divers coming from outside Sicily. Images of *A. calycularis* are iconic symbols of the diving attractions in the websites of those diving centers (source: the Egadi Archipelago diving centers).

Human pressure on coastal areas is increasing, with severe impacts on marine biodiversity^3^. Thus field studies like this one, providing baseline information on the status of endangered species and the effect of anthropogenic impacts on them, are crucial to achieve proper information for their management and conservation. The shallow-water sites hosting *A. calycularis* bioconstructions are generally small coves and cliffs not larger than a few hundred square metres^32^. Properly managed marine protected areas have strong potential to prevent damage from intense use of coastal habitats. However, as already suggested^32^, the creation of microprotected areas allowing specific restrictions and ensuring proper communication to the large public could be the best practice to achieve the conservation of coastal bioconstructions. Non protected small coves and cliffs where the orange coral usually thrives appear to be good candidates to test and implement focused protection schemes.

## Supplementary information

Dataset 1

## Data Availability

The datasets generated and/or analysed during the current study are available from the corresponding author on reasonable request.
