# Supplementary material for: Anthropogenic impact is negatively related to coral health in Sicily (Mediterranean Sea)
Source: Sci Rep. 2019 Sep 17;9:13469. doi: 10.1038/s41598-019-49713-w (PMC6748980; doi:10.1038/s41598-019-49713-w)
Supplement: Supplementary file 1 — Dataset 1 [file 41598_2019_49713_MOESM1_ESM.pdf]

Anthropogenic impact is negatively related to coral health in Sicily (Mediterranean Sea)

Fiorella Prada[a†], Luigi Musco[b†*], Adriana Alagna[b], Davide Agnetta[c], Eleonora Beccari[a], Giovanni D'Anna[d], Vincenzo Maximiliano Giacalone[e], Carlo Pipitone[d], Tomás Vega Fernández[b], Stefano Goffredo[a], Fabio Badalamenti[d, b, f]

[a] Marine Science Group, Department of Biological, Geological and Environmental Sciences, University of Bologna, Via F. Selmi 3, 40126 Bologna, Italy

[b] Stazione Zoologica Anton Dohrn, Integrative Marine Ecology Department, Villa Comunale, 80121 Naples, Italy

[c] Istituto Nazionale di Oceanografia e di Geofisica Sperimentale, Via Beirut 2/4, 34151 Trieste, Italy

[d] Consiglio Nazionale delle Ricerche, Istituto per lo studio degli impatti Antropici e Sostenibilità in ambiente marino, Via Giovanni da Verrazzano, 17 - 91014 Castellammare del Golfo, Italy

[e] Consiglio Nazionale delle Ricerche, Istituto per lo studio degli impatti Antropici e Sostenibilità in ambiente marino, Via del mare 3, - 91021 Torretta Granitola, Italy

[f] School of Geosciences, Grant Institute, King's Buildings, University of Edinburgh, Edinburgh, United Kingdom

SUPPLEMENTARY TABLES

Table S1. Values of the D statistic and its associated probability for the comparisons of the shapes of the polyp size frequency distributions between pairs of localities along a gradient of human impact. Sample sizes indicated in column n. Bold numbers indicate significant differences at a nominal alpha level of 0.05. Loc 1 = Marettimo; Loc 2 = Favignana; Loc 3 = Zingaro; Loc 4 = Capo Gallo; Loc 5 = Capo Zafferano.

Location	n	Loc 1	Loc 2	Loc 3	Loc 4	Loc 5
**Loc 1**	642					
**Loc 2**	442	0.05 (>0.100)				
**Loc 3**	449	**1.17 (<0.001)**	**0.22 (<0.001)**			
**Loc 4**	300	**0.53 (<0.001)**	**0.51 (<0.010)**	**0.39 (<0.001)**		
**Loc 5**	535	**0.26 (<0.001)**	**0.31 (<0.001)**	**0.09 (<0.050)**	**0.44 (<0.010)**	

Table S2. Cumulative (I_C), Land-based (I_L) and Sea-based (I_S) human impact at five locations. Loc 1 = Marettimo; Loc 2 = Favignana; Loc 3 = Zingaro; Loc 4 = Capo Gallo; Loc 5 = Capo Zafferano.

	Loc 1	Loc 2	Loc 3	Loc 4	Loc 5
I_C	8.50	11.23	26.39	28.22	32.59
I_L	0.00	2.39	16.96	17.60	23.56
I_S	8.5	8.82	9.43	10.62	9.03

Table S3. Average sea surface temperature (SST AVG) and average annual variation of sea surface temperature (SST VAR) at the five analyzed locations over five years (2009 to 2013). Data extracted from the NASA online database Giovanni. Loc 1 = Marettimo; Loc 2 = Favignana; Loc 3 = Zingaro; Loc 4 = Capo Gallo; Loc 5 = Capo Zafferano.

	Loc 1	Loc 2	Loc 3	Loc 4	Loc 5
**SST AVG**	19.78	19.43	20.22	20.38	20.55
**SST VAR**	12.87	12.00	12.96	13.33	13.51

Table S4. Pearson correlation (-1 to 1) among the predictor variables tested in the DISTLM-forward non-parametric multiple regression analyses. Cumulative (I_C). Land-based (I_L). Sea-based (I_S) human impact. average sea surface temperature (SST AVG). average annual variation of sea surface temperature (SST VAR)

	I_C	I_L	I_S	SST AVG	SST VAR
I_C	/				
I_L	0.998	/			
I_S	0.611	0.560	/		
**SST AVG**	0.934	0.934	0.551	/	
**SST VAR**	0.777	0.778	0.447	0.949	/

Table S5. **Distribution of *Astroides calycularis* polyp size classes in the five analyzed locations.** Average and median polyp length (mm). skewness. modal class (mm) and number of classes at each location. Loc 1 = Marettimo; Loc 2 = Favignana; Loc 3 = Zingaro; Loc 4 = Capo Gallo; Loc 5 = Capo Zafferano.

Location	n	Mean	Median	Mode	Min.	Max.	S.D.	Skewness	Kurtosis
**Loc 1**	641	7.51	7	6	4	13	1.98	0.42	-0.44
**Loc 2**	442	7.47	7	6.9	3	12	1.94	0.14	-0.82
**Loc 3**	449	6.6	7	6	3	11	1.62	0.12	-0.59
**Loc 4**	300	5.49	6	6	2	9	1.01	0.04	0.54
**Loc 5**	535	6.58	7	7	3	10	1.29	-0.40	-0.29

Table S6. Land-based and sea-based stressors. Values of weighting variables and stressors at the five study locations [values of stressors extracted from the georeferenced layers developed by Micheli et al. (2013) and rescaled between 0 and 1]. Loc 1 = Marettimo; Loc 2 = Favignana; Loc 3 = Zingaro; Loc 4 = Capo Gallo; Loc 5 = Capo Zafferano.

**Driver of change**	**Weighting variable (expert judge between 0 and 10)**		**Stressor value scaled between 0 and 1**				
	**average**	**S.D.**	**Loc 1**	**Loc 2**	**Loc 3**	**Loc 4**	**Loc 5**
**Artisanal fisheries**	1.60	1.34	0.00	0.08	0.09	1.00	0.28
**Non-destructive demersal fisheries high by-catch**	1.00	1.22	1.00	0.35	0.36	0.58	0.52
**Non-destructive demersal fisheries low by-catch**	0.50	1.00	0.60	0.72	0.26	0.50	1.00
**Destructive demersal fisheries**	2.00	1.63	0.74	0.76	0.38	0.62	1.00
**Pelagic fisheries**	1.00	1.41	0.83	1.00	0.48	0.63	0.73
**Invasive species**	4.75	2.22	0.71	1.00	1.00	0.86	0.86
**Shipping**	2.80	1.64	0.53	0.27	1.00	0.80	0.27
**Pesticides**	7.00	1.22	0.00	0.09	1.00	0.20	0.47
**Population**	8.20	1.30	0.00	0.08	0.09	0.96	1.00
**Fertilizers**	7.40	1.52	0.00	0.11	1.00	0.25	0.55
**Urban run-off**	8.00	1.00	0.00	0.03	0.23	0.80	1.00
